# Report of five cases: sandwich repair for post infarction ventricular septal rupture with right ventricular approach

**DOI:** 10.1186/s43044-020-00048-2

**Published:** 2020-03-16

**Authors:** Shahyad Salehi-ardebili, Hamid Mehdizade, Behnam Askari

**Affiliations:** Department of Cardiovascular Surgery, Seyyed-al-Shohada Heart Center, Urmia University of Medical Sciences, 17 Shahrivar Street, Urmia, Iran

**Keywords:** Post-infarction ventricular septal rupture, Right ventricle, Sandwich technique, Case report

## Abstract

**Background:**

Different techniques have been described to repair post myocardial infarction ventricular septal rupture (VSR), each method may result in residual shunting, bleeding, and suboptimal left ventricular (LV) performance secondary to change in LV geometry. The aim of this report is to describe early and mid-term results of sandwich technique through right ventricle in five consecutive patients.

**Case presentation:**

Five consecutive patients (3 women and 2 men) with VSR (mean age 62.8 years, range 51-70) underwent surgical repair for postinfarction ventricular septal rupture by sandwich technique performed through right ventricle from August 2012 to April 2019 in our institute. Reconstruction of the septum was performed by two patches of 0.6 mm Gore-Tex on each side of the septal defect through right ventricular incision, according to the method described by Isoda et al. Coronary artery bypass grafting was performed in two patients.

The mean aortic clamp time was 90 min (range, 64 to 157 min). The mean extracorporeal circulation time was 146.6 min (range, 108 to 240 min). Postoperative intensive care unit (ICU) stay averaged 12 days (range, 4-40 days). There was no hospital mortality. No postoperative residual shunting was detected, and no patient needed re-operation for bleeding. Patients have been followed up for a mean of 24.4 months (range, 1 week to 7 years). There was one death seven days after discharge due to arrhythmia (40 days after surgery).

**Conclusion:**

Sandwich technique through right ventricular approach is simple and extendable to all VSRs irrespective of their locations. Residual shunting and bleeding are negligible or zero. It may be considered as standard of repair for patient with post infarction ventricular septal rupture.

## Background

Ventricular septal rupture (VSR) is a rare but lethal complication of myocardial infarction. It complicates less than 0.5% of patients following acute myocardial infarction [1]. Medical therapy alone bears an unacceptably high mortality, for example, a mortality of 96% (one survival among 24) was reported from SHOCK trial registry [2].

Many surgical techniques have been described including infarctectomy and closure (Daggett’s technique), infarct exclusion and its modifications (single or double patch) with or without application of surgical glue [3], and sandwich technique. Ideally, the repair should result in no residual shunting, be reproducible and maintain left ventricular geometry.

Many current techniques do not fulfill this criterion. Isoda et al. have developed a method known as sandwich technique that effectively addresses these problems [4]. They used right ventriculotomy with double patch and surgical glue to close VSR. No significant leaks, no early mortality, and no post-operative bleeding occurred among their seven consecutive patients.

Based on the encouraging results achieved by Isoda technique, we operated five consecutive patients with the diagnosis of VSR using this method and here we reported the results.

## Case presentation

We reviewed five consecutive patients with postinfarction ventricular rupture who referred to us from August 2012 to April 2019 for surgical repair at Seyed-al-Shohada Heart Hospital, Urmia, Iran. Steps of the operation were the same for all patients.

All patients were supported with intra-aortic balloon pump (IABP) immediately after the diagnosis. The operations were performed on cardiopulmonary bypass and bicaval cannulation. Venous line de-airing was routinely undertaken. Arterial filter (Eurosets) and pre-bypass filter (Eurosets EU 3709) were routinely used. Affinity (3 cases) and Dideco Evo Oxygenators (2 cases) were used. Moderate hypothermia (28 C) was used. During CPB, both lungs were ventilated with 150-200 mL of air to prevent atelectasis. First dose of cardioplegia administered initially into the aortic root as warm induction until heart was arrested and then the rest of it were given through coronary sinus. Next doses of cardioplegia were administered only through coronary sinus.

During cardiac arrest, intra-aortic balloon pump (IABP) was set to internal mode to make the flow pulsatile. The operation field was flooded with CO2 to lessen the chance of air emboli. At the conclusion of the operation, warm shot was infused into the coronary sinus, followed by controlled aortic root reperfusion.

**Fig. 1:**
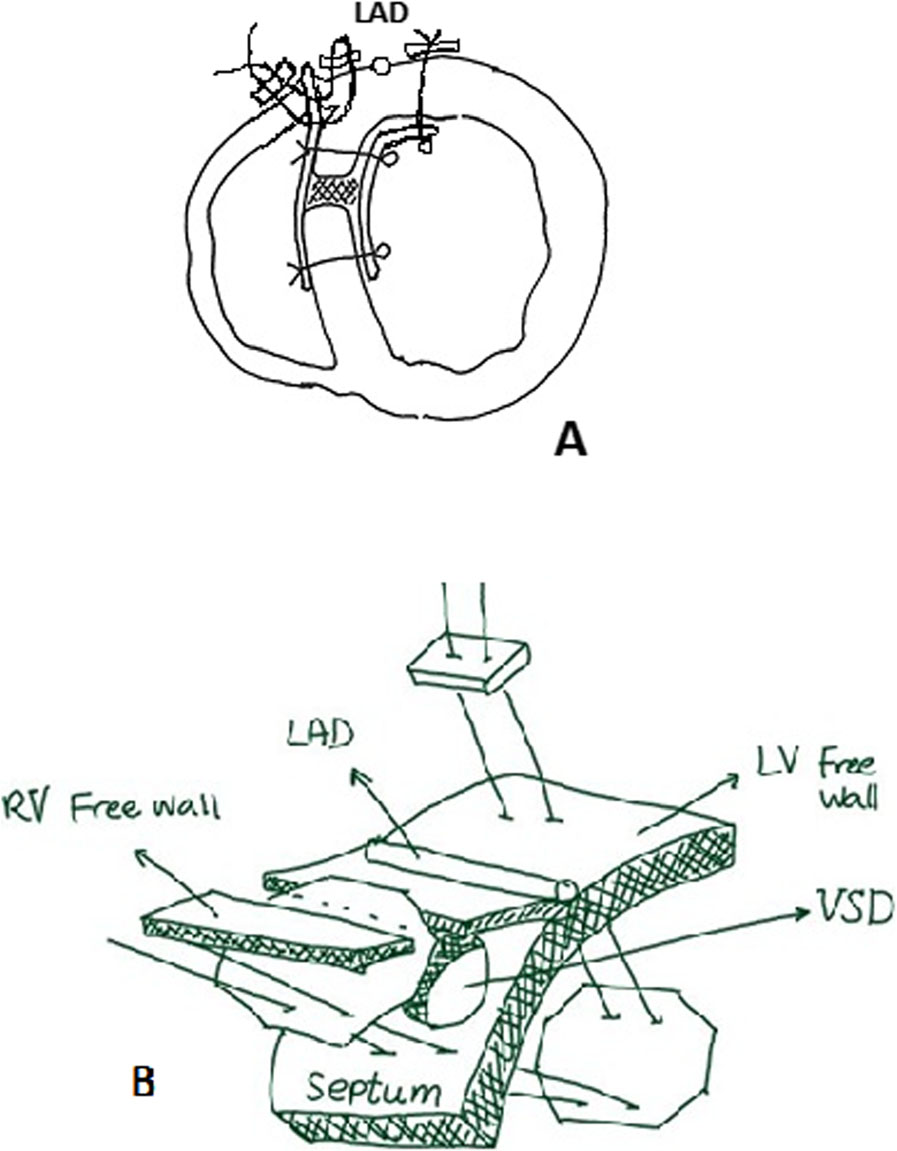
Sandwich technique with use of biological glue

Surgical technique, right ventricle (RV) was opened about 1.5 to 2 cm lateral and parallel to the left anterior descending coronary artery (LAD) about 5 or 6 cm to fully visualize VSD. In order to expose septal defect, trabeculae were widely cut. Two patches of 0.6 mm Gore-Tex measuring approximately 5 in 5 cm were trimmed at their corners to make them octagon. On average about 10-11 sutures of “25 mm 3-0 prolene” were used, 6 or 7 to sandwich the septum between the patches. These sutures were placed in the following order: left ventricular Gore-Tex patch, LV side of the septum emerging from right ventrivilar side of the septum then to the second (right ventricular) patch. The remaining 4 or 5 sutures were used to anchor LV patch to free wall of the LV about 1.5 cm to the left of LAD being secured with Teflon felts pledgets. Then LV patch was pushed throgh septal defect into the LV cavity. RV patch at its free wall is left intact to be later incorporated with sutures for closing the RV incision with two strip of Teflon felt (Fig. 1). The remaining of operation was as routine.

Preoperative data are displayed in Table 1. The mean age of patients was 62.8 years (range from 51 to 70). In all patients, septal defect was located at the anterior wall. The duration from the onset of the infarction to the operation was 3 to 28 days (mean 15.8 days). Operative and postoperative data are displayed in Table 2.

**Table 1:** Preoperative data

Variable	Mean (range)
Gender	females 3, male 2
Preoperative EF	24.8% (20-30%)
Age	62.8 years (51-70 years)
IABP to operation	8.4 days (2-11 days)
Intubated preoperatively	2 of 5 days
Site	Anterior in all

**Table 2:** Operative and postoperative data

Variables	Mean (range)
Aortic cross clamp	*90 min* (*64-157 min*)
CPB-time	*147 min* (*108-240 min*)
CABG	*2 of 5*
Postoperative EF	*24%* (*20-30%*)
Post-operation intubation	*6.3 days* (*6 h-30 days*)
Postoperative IABP	*2.8 days* (*1-5 days*)
Postoperative ICU stay	*11.8 days* (*4-40 days*)
Bleeding	*0 patients* (*n = 5*)
Residual shunting	*0 patients*
In-Hospital mortality	*N0*
Long term survival	*4 patients* (*n = 5*)

Two patients received a simultaneous coronary artery bypass graft (CABG). In one case saphenous vein graft (SVG) was grafted to obtuse marginal (OM), and in the other one SVG was grafted to OM and posterior descending artery (PDA). In neither case, LAD was graftable. The mean aortic clamp time was 90 min (range of 64 to 157 min). The mean cardiopulmonary bypass (CPB) time was 146.6 min (range of 108 to 240 min).

The mean postoperative use of IABP was 2.8 days (range 1 to 5 days). Postoperatively, no residual shunting was detected by echocardiographic study. There was no hospital mortality.

One patient died of arrhythmia 40 days after surgery and 7 days after hospital discharge. This case was a 69-year-old diabetic woman received at our center in cardiogenic shock with EF of 20%. IABP was placed and while stabilizing the patient she experienced cardiac arrest due to ventricular fibrillation and underwent resuscitation (CPR), tracheal intubation, and mechanical ventilation. Subsequently, she developed disseminated intravascular coagulation (DIC) and renal failure with creatinine levels reaching 4 mg/dL. After controlling arrhythmia and some improvement in her condition, she underwent surgery. Postoperatively, she had very complicated course of arrhythmia, low cardiac output, pressure ulcers, generalized edema, and respiratory failure; with supportive measures, she survived to discharge with a total hospital stay of 52 days. One week following hospital discharge, she died of arrhythmia. The remaining (80%) are currently alive and in NYHA functional class 2.

## Conclusion

Before the introduction of thrombolytic therapy, 1% to 2% acute myocardial infarctions (MI) were complicated with ventricular septal defect (VSD), so it is fairly uncommon [5]. With medical therapy, about 25% of patients in cardiogenic shock die within 24 h and 50% or more do so within 1 week after developing septal rupture [6].

Timing of operation may be depended on patient’s condition. Patients in cardiogenic shock (50 to 60% of patients) need immediate surgery. Delaying treating patients in cardiogenic shock represents a “failed strategy.” Stable patients represent less than 5% of total, surgery in this group may be safely delayed, allowing necrotic tissue become fibrotic. Patients with intermediate position between shock and stable condition. These patients should be operated on promptly within 12 to 24 h [6, 7].

There are four main surgical techniques to repair VSR, including infarctectomy and closure (Daggett’s technique), infarct exclusion, modified infarct-exclusion technique double patch plus biological glue, and sandwich technique through right ventricular approach.

**Fig. 2:**
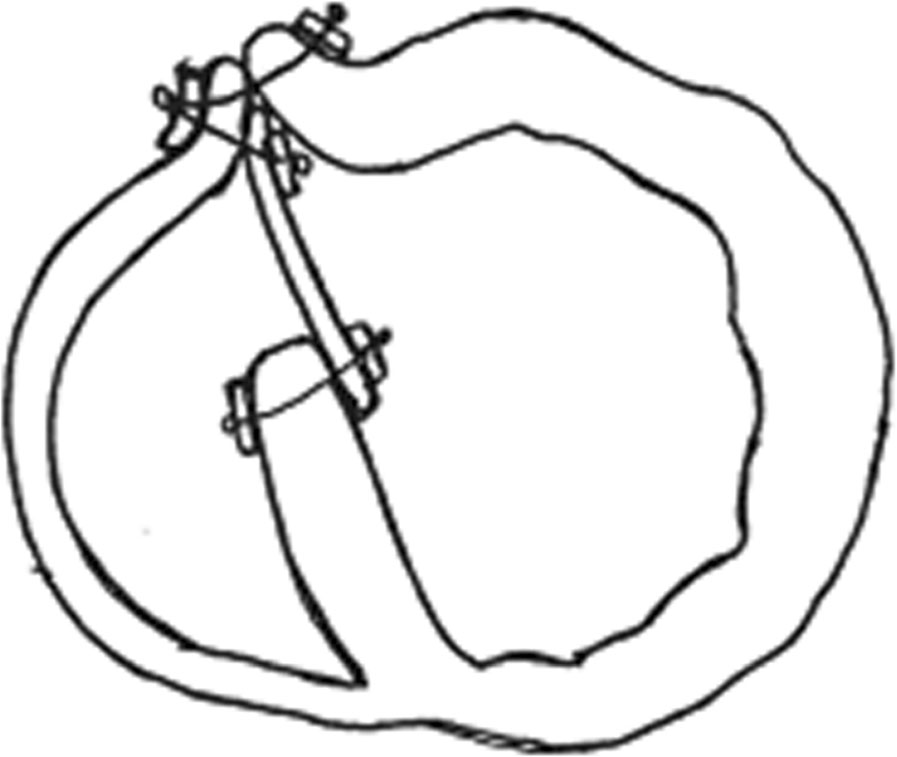
Daggett’s infarctectomy and septal rupture reconstruction

Furukawa et al. reported 12 patients who underwent Daggett’s infarctectomy and septal rupture reconstruction with Dacron patch from 1990 to 1998 with no in-hospital mortality. Coronary artery bypass grafting was performed in 5 patients. In their series, postoperative residual shunt was recognized in 3 patients (25%); none of them was hemodynamically significant, so re-operation was not needed. They reported two late deaths caused by non-cardiac problems [8] (Fig. 2). Considerable tension at the suture with potential risk of residual shunting and bleeding, reduction in left ventricle size, and change in the geometry are the main concerns when Daggett’s technique is used.

**Fig. 3:**
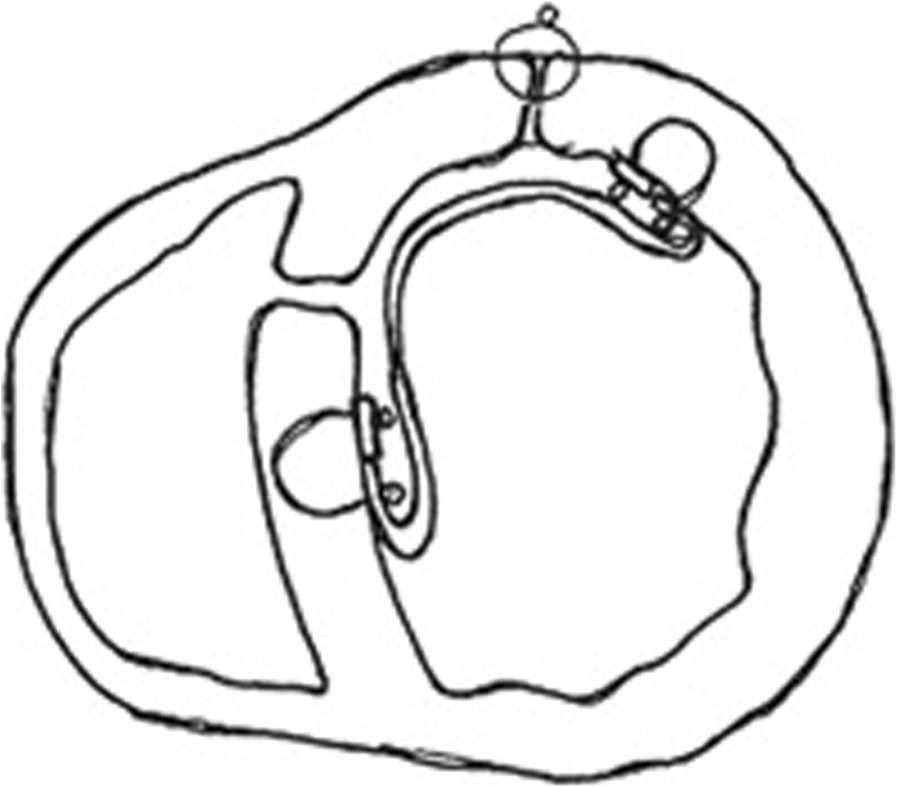
Komeda type infarct exclusion technique

The Komeda type exclusion technique through the left ventricular cavity avoids stitching necrotic myocardium, so minimal or no infarctectomy is needed. A single patch of bovine pericardium is sutured to non-necrotic-ischemic tissue. The right ventricle is not incised. Residual shunting is still a concern and potential change in LV geometry remains, although tearing of the fragile ischemic myocardium may be prevented [9] (Fig. 3).

**Fig. 4:**
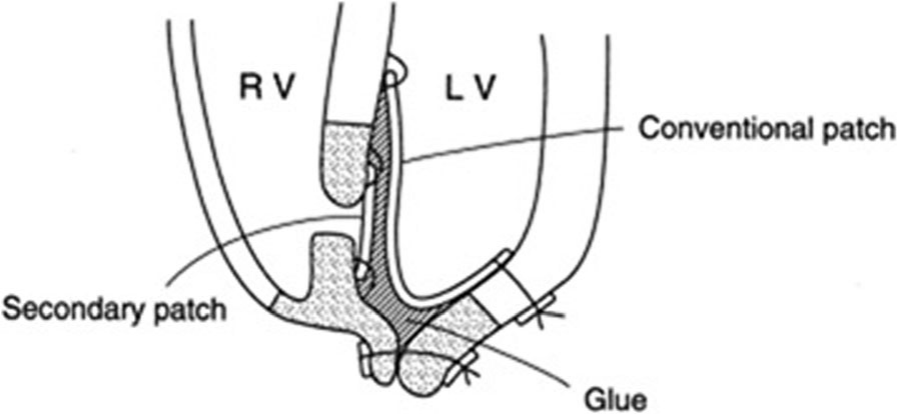
Modified infarct-exclusion technique with double patch and application of biological glue

Residual leak is a common problem after surgical repair for VSR. Deja et al. reported this complication in 40% of patients with different surgical techniques. In their study, reoperation was needed in 12% of patients and operative mortality was 35% [10]. Residual leak may be prevented using the modified infarct-exclusion technique with double patch and application of biological glue, but it is technically demanding and change in LV geometry may still be a problem [11] (Fig. 4).

Isoda et al. introduced a method known as the sandwich technique in which septum is sandwiched between two patches of Gore-Tex anchored together (Fig. 1). Table 3 compares some aspects of various repairs.

**Table 3:** Characteristics of common methods for surgical repair of post-infarction ventricular septal rupture

Technique	Infarctectomy and closure (Daggett’s technique)	Infarct exclusion	Modified infarct-exclusion technique double patch plus biological glue	Sandwich technique
Septal patch	Single	Single	Double	Double
Approach	Left ventricle	Left ventricle	Left ventricle	Right ventricle
Reproducibility	Difficult	Difficult	Difficult	Simple
Residual shunting	Frequent	Uncommon	Rare	Rare
Preservation of left ventricular geometry	Poor	Poor	Poor	Good

We performed this technique in five consecutive patients. Surgical glue was applied in only one case, although Isoda recommended glue to lessen stress at suture [4]. No patient is put on warfarin postoperatively. Thromboembolic events did not occur.

Approach from RV is superior than LV because LV geometry is better preserved; mitral valve apparatus is less likely to be damaged; bleeding is of lesser concern due to lower pressures of RV; and the septal defect is better visualized, making it easier for placing the patches.

The principles of the repair of posterior VSR is quite similar to anterior ones. The incision is about 1-1.5 cm from and parallel to posterior descending artery, large enough to fully visualize the septal defect. It is important to resect obscuring trabeculae [12].

This technique is simple and reproducible, and the LV geometry is not changed. We experienced no residual shunting, no reoperation, and no operative death. In our experience, no significant right heart failure was developed secondary to right ventriculotomy.

## Data Availability

Available upon request

## Abbreviations

VSR: Ventricular septal rupture
LV: Left ventricular
ICU: Intensive care unit
IABP: Intra-aortic balloon pump
CPB: Cardiopulmonary bypass
RV: Right ventricle
LAD: Left anterior descending
VSD: Ventricular septal defect
CABG: Coronary artery bypass graft
SVG: Saphenous vein graft
OM: Obtuse marginal
PDA: Posterior descending artery
CPR: Cardiopulmonary resuscitation
DIC: Disseminated intravascular coagulation
NYHA: New York Heart Association
